# Effects of Ga and Si Incorporation on Oxygen-Related Defects and Bias-Temperature Stability of ZnSnO Thin-Film Transistors

**DOI:** 10.3390/mi17080985

**Published:** 2026-08-21

**Authors:** Sang Ji Kim, Jaehong Park, Wonjun Shin, Sang Yeol Lee

**Affiliations:** 1 Department of Semiconductor Engineering, Gachon University, Seongnam-si 13120, Republic of Korea; kej5657962@gachon.ac.kr; 2 Gachon Advanced Institute of Semiconductor Technology, Gachon University, Seongnam-si 13120, Republic of Korea; 3 Department of Semiconductor Convergence Engineering, Sungkyunkwan University, Suwon 16419, Republic of Korea; qkrwoghd789@skku.edu; 4 Department of Electrical and Computer Engineering, Sungkyunkwan University, Suwon 16419, Republic of Korea

**Keywords:** Zn–Sn–O, thin-film transistor, dopant incorporation, oxygen vacancy, Urbach energy, bias-temperature stability

## Abstract

Zn–Sn–O (ZTO) thin-film transistors (TFTs) are promising indium-free oxide semiconductor devices, but their electrical stability is limited by oxygen-related defect states. In this study, Ga and Si incorporated ZTO TFTs were systematically compared using an identical bottom-gate top-contact device architecture to investigate dopant-dependent defect modulation and bias-temperature stability. Both Ga and Si incorporation induced a positive threshold-voltage shift and reduced the relative contribution of oxygen-deficient bonding components, suggesting modification of oxygen-related defect environments in the ZTO channel. Optical analysis further showed reduced Urbach energies after dopant incorporation, suggesting a decrease in localized band tail states and reduced structural disorder. Under negative bias temperature stress (NBTS), SZTO exhibited the smallest threshold-voltage shift, demonstrating the most effective stability enhancement. These results indicate that Ga incorporation preserves high field-effect mobility while improving stability, whereas Si incorporation more effectively reduces oxygen-related defect features and provides enhanced NBTS stability. This study provides insight into the dopant-dependent defect engineering for the improved reliability of indium free oxide TFTS.

## 1. Introduction

Thin-film transistors (TFTs) based on amorphous oxide semiconductors (AOSs) have attracted considerable attention for next-generation display and electronic applications owing to their high optical transparency, low-temperature processability, and excellent electrical performance [1,2,3]. Among various oxide semiconductor materials, indium–gallium–zinc oxide (IGZO) has been widely employed as an active channel material owing to its relatively high field-effect mobility, low off-state current, and good large-area uniformity [4,5,6]. However, concerns regarding the cost, supply concentration, and long-term availability of indium have motivated extensive research into indium-free oxide semiconductors [7,8].

Zn–Sn–O (ZTO) has emerged as a promising indium-free candidate because it can provide relatively high carrier mobility while avoiding the use of indium [9,10,11]. Nevertheless, ZTO TFTs can exhibit pronounced electrical instability under illumination and photo-bias stress owing to oxygen-related defect states. Oxygen-deficient configurations can contribute to donor-like states and charge-trapping sites, resulting in variations in carrier concentration, threshold-voltage shifts, and degraded device reliability [12,13,14,15]. Therefore, suppressing oxygen-related defect states while improving bias-temperature stability without excessively sacrificing field-effect mobility remains a critical challenge for reliable ZTO-based TFTs.

Dopant incorporation is an effective strategy for regulating oxygen-related defects and carrier concentration in amorphous oxide semiconductors. Among candidate dopants, gallium (Ga) and silicon (Si) are attractive carrier suppressors owing to their strong affinity for oxygen [16,17,18]. At moderate concentrations, Ga incorporation has been reported to suppress oxygen-deficiency-related carrier generation with a relatively small mobility penalty in amorphous oxide semiconductors [18,19]. In contrast, Si incorporation has been reported to provide effective carrier suppression owing to its strong oxygen-binding characteristics, although excessive Si incorporation may reduce field-effect mobility by limiting carrier transport [20,21]. Thus, previous studies suggest that Ga and Si can provide different trade-offs between carrier transport and defect suppression; however, these effects have largely been examined in separate dopant systems.

Systematic side-by-side comparisons of Ga- and Si-incorporated ZTO under identical material-processing and device conditions remain limited. In particular, it remains unclear how the two dopants differently influence oxygen-related chemical environments, energetic disorder, carrier transport, and bias-temperature stability when evaluated within the same TFT platform. In this work, ZTO-, GZTO-, and SZTO-based TFTs were fabricated using an identical bottom-gate, top-contact device structure and the same processing conditions. The dopant-dependent evolution of oxygen-related bonding environments and optical band-tail states was examined using O 1s XPS analysis and Urbach-energy analysis, respectively, and correlated with the electrical characteristics and negative-bias-temperature-stress stability of the devices. By directly comparing these three channel materials within a common experimental framework, this study clarifies the relative effects of Ga and Si incorporation on the defect/carrier balance, energetic disorder, and operational stability of indium-free ZTO TFTs.

## 2. Experimental Details

**Figure 1:**
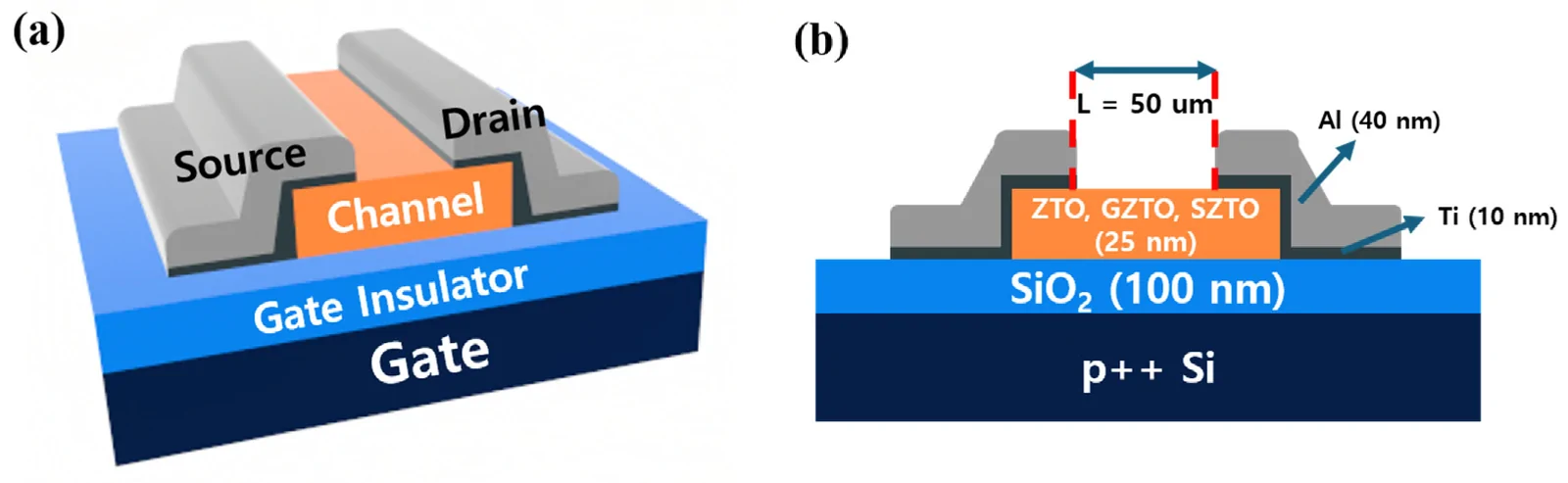
(**a**) Three-dimensional schematic illustration of the fabricated ZTO-, GZTO-, and SZTO-based TFTs, and (**b**) cross-sectional schematic of the bottom-gate, top-contact TFT structure.

Figure 1a shows a three-dimensional schematic illustration of the fabricated ZTO, GZTO, and SZTO based TFTs, while Figure 1b presents the corresponding cross-sectional structure of the bottom-gate top-contact TFT. Heavily doped p-type Si wafers were used as the gate electrode, and a 100-nm-thick SiO_2_ layer served as the gate insulator. Prior to film deposition, the substrates were sequentially cleaned in acetone, methanol, and deionized water using ultrasonic agitation for 10 min each, followed by drying with nitrogen gas.

ZTO, GZTO, and SZTO thin films were deposited as active channel layers by RF magnetron sputtering at room temperature using separate ceramic targets. The ZTO target had a Zn:Sn composition ratio of 65:35. To investigate the effects of dopant incorporation, GZTO and SZTO targets with the same Zn:Sn ratio and nominal dopant concentrations of 0.5 wt% Ga and 0.5 wt% Si respectively, were prepared. The sputtering process was carried out under an Ar atmosphere with a flow rate of 40 sccm, a working pressure of 12 mTorr, and an RF power of 50 W. The target-to-substrate distance was 20 cm, the base pressure prior to deposition was below 6.0 × 10^−6^ Torr, and the deposition rate was approximately 5.5 nm/min. The channel thickness was controlled to approximately 25 nm. After channel patterning using a lift-off process, the deposited films were annealed in ambient air at 500 °C for 2 h.

For the source and drain electrodes, Ti/Al bilayers were deposited by sequential electron-beam evaporation and thermal evaporation. A 10-nm-thick Ti layer was used as the adhesion layer, and a 40-nm-thick Al layer was used as the conductive electrode. The electrodes were then defined through a lift-off process. The fabricated TFTs had a channel width and length of 250 and 50 μm, respectively.

The electrical characteristics of the fabricated TFTs were measured using a semiconductor parameter analyzer (EL423, ELECS Co., Ltd., Goyang, Republic of Korea). For each composition, three TFTs were characterized, with one device obtained from each of three independently fabricated batches. The extracted electrical parameters are reported as the mean ± standard deviation. The surface morphology, chemical bonding states, and optical properties of the channel films were investigated by atomic force microscopy (AFM; NX10, Park Systems Corp., Suwon, Republic of Korea), X-ray photoelectron spectroscopy (XPS; Nexsa G2, Thermo Fisher Scientific, East Grinstead, UK), and UV–visible spectroscopy (Cary 5000, Agilent Technologies, Santa Clara, CA, USA) respectively. For XPS analysis, a monochromated, micro-focused Al Kα X-ray source with a photon energy of 1486.6 eV was used. High-resolution spectra were acquired using a 300 μm X-ray spot, a pass energy of 20 eV, and an energy step of 0.05 eV, with a dual-beam flood gun employed to minimize sample charging. The optical bandgap was extracted from Tauc plots, and the Urbach energy was obtained from the absorption edge region. Negative bias temperature stress (NBTS) measurements were conducted to evaluate the bias-temperature stability of the ZTO, GZTO, and SZTO based TFTs.

All fabrication and measurement conditions were kept identical except for the channel composition to ensure that the observed differences originated primarily from Ga and Si incorporation.

## 3. Results and Discussion

**Figure 2:**
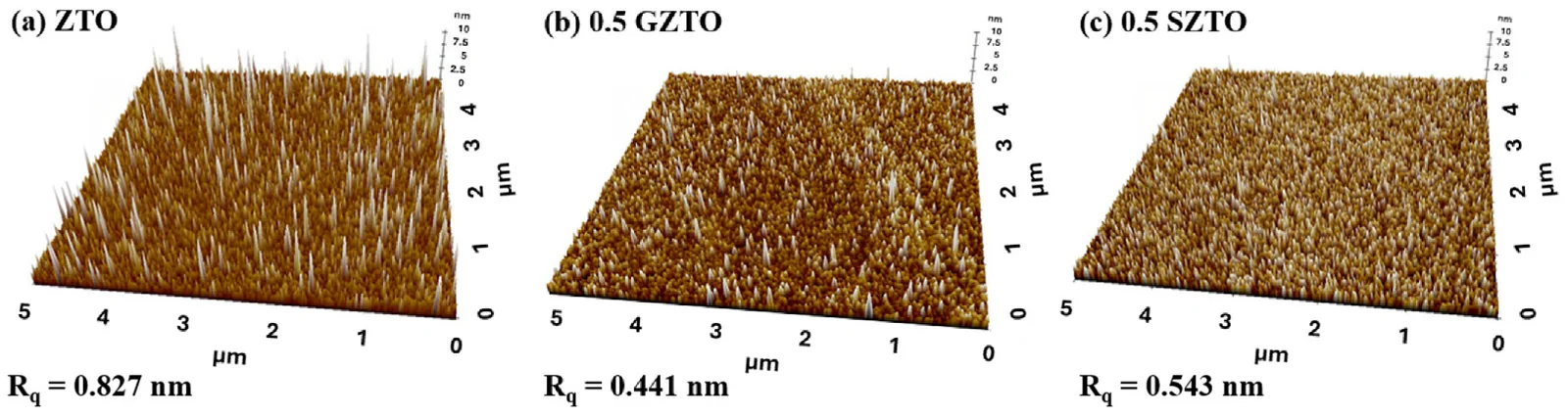
AFM surface morphology images of the (**a**) ZTO, (**b**) GZTO, and (**c**) SZTO channel layers. The extracted root-mean-square roughness (R_q_) values decreased from 0.827 nm for ZTO to 0.441 and 0.543 nm for GZTO and SZTO, respectively, indicating improved surface uniformity after Ga and Si incorporation.

Figure 2 shows the AFM surface morphology images of the ZTO, GZTO, and SZTO channel films. The ZTO film exhibited a root-mean-square roughness (R_q_) of 0.827 nm, whereas the R_q_ values decreased to 0.441 nm and 0.543 nm for the GZTO and SZTO films, respectively. All films showed smooth surfaces with sub-nanometer roughness, indicating that uniform channel layers were formed under the present sputtering conditions.

The reduced R_q_ values of the GZTO and SZTO films suggest that Ga and Si incorporation can influence the film-growth behavior of ZTO-based oxide semiconductors. In particular, the GZTO film exhibited the lowest R_q_ value, indicating improved surface uniformity. Since all films exhibited sub-nanometer roughness, the differences in electrical stability discussed below are unlikely to originate primarily from severe morphological degradation. Therefore, the following discussion focuses on dopant-induced defect modulation rather than surface roughness effects.

**Figure 3:**
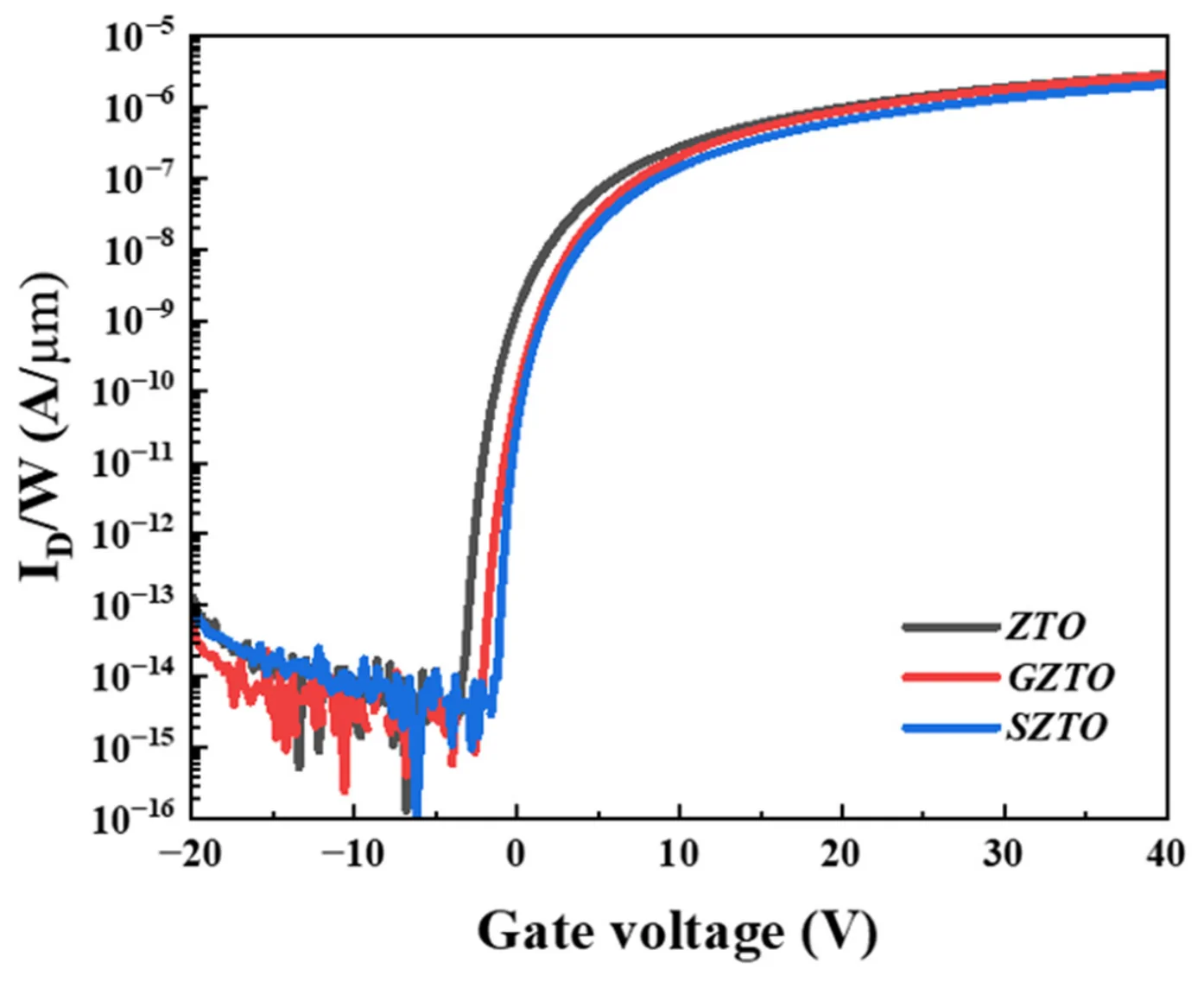
Transfer characteristics of the ZTO, GZTO, and SZTO based TFTs, showing the positive shift in threshold voltage depending on Ga and Si incorporation.

Figure 3 shows representative transfer characteristics of the ZTO, GZTO, and SZTO TFTs. The corresponding electrical parameters obtained from three independently fabricated devices for each composition are summarized in Table 1 as the mean ± standard deviation. The transfer characteristics were measured under ambient conditions at a fixed drain voltage of V_DS_ = 5 V while sweeping the gate voltage from V_GS_ = −20 to 40 V. The threshold voltage (V_TH_) was determined by linear extrapolation of the linear portion of the I_DS_–V_GS_ transfer characteristics to the V_GS_ axis. The field-effect mobility (μ_FE_) was extracted from the transconductance in the linear regime, and the subthreshold swing (S.S.) was determined from the inverse slope of the transfer characteristics in the subthreshold region. All devices exhibited typical n-type switching behavior with high on/off current ratios on the order of 10^8^, confirming reliable TFT operation for all channel compositions.

**Figure 4:**
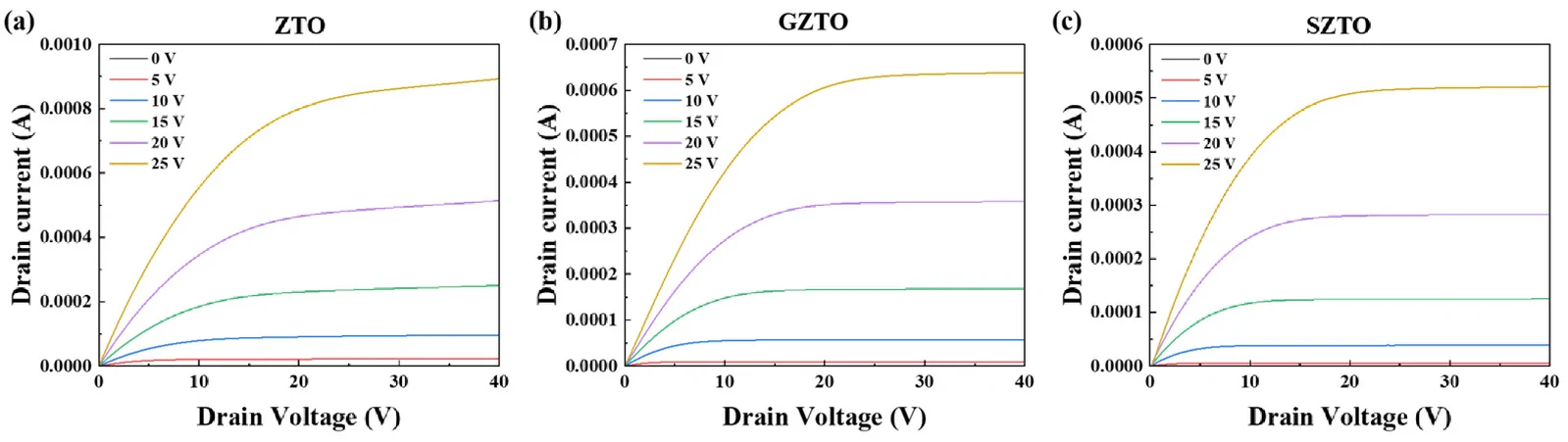
Output characteristics of (**a**) ZTO, (**b**) GZTO, and (**c**) SZTO TFT.

Figure 4 presents the corresponding output characteristics of the ZTO, GZTO, and SZTO TFTs measured at different gate voltages. All devices exhibited a linear increase in drain current at low drain voltage, followed by clear current saturation at higher drain voltage, confirming typical n-channel TFT operation. Consistent with the transfer characteristics, ZTO and GZTO showed comparable current-driving capability, whereas SZTO exhibited a relatively lower drain current, reflecting the stronger carrier suppression induced by Si incorporation.

The ZTO TFTs exhibited an average V_TH_ of −2.48 ± 0.41 V, whereas the average V_TH_ values of the GZTO and SZTO TFTs shifted positively to −1.32 ± 0.55 and −0.61 ± 0.48 V, respectively. This positive V_TH_ shift indicates that Ga and Si incorporation modified the threshold-voltage position of the ZTO-based TFTs. In addition, the average μ_FE_ values of the ZTO and GZTO TFTs were 27.1 ± 0.35 and 27.6 ± 0.43 cm^2^ V^−1^ s^−1^, respectively, while the SZTO TFTs showed a lower average μ_FE_ of 21.6 ± 0.61 cm^2^ V^−1^ s^−1^. These results indicate that Ga and Si incorporation affected carrier transport differently. The comparable μ_FE_ values of ZTO and GZTO show that Ga incorporation did not significantly degrade carrier transport despite the positive V_TH_ shift and slight reduction in I_ON_. This suggests that the moderate carrier suppression induced by Ga incorporation was not accompanied by a substantial deterioration of the transport pathways.

The average subthreshold swing (S.S.) values were 0.465 ± 0.012, 0.461 ± 0.018, and 0.437 ± 0.010 V dec^−1^ for the ZTO, GZTO, and SZTO TFTs, respectively. A slightly lower average S.S. was observed for the doped devices, particularly for the SZTO TFTs, although the differences were relatively modest considering the observed device variation. In amorphous oxide semiconductors, carrier transport is generally governed by trap-limited and percolative conduction, where higher carrier concentrations facilitate localized-state filling and the formation of effective conduction pathways [22,23]. In contrast to GZTO, the reduced μ_FE_ of SZTO, together with its stronger positive V_TH_ shift and lower I_ON_, suggests that the mobility decrease is more likely associated with stronger carrier suppression and reduced percolative transport efficiency rather than with increased defect scattering.

Overall, the transfer characteristics show that Ga and Si incorporation changed the electrical properties of ZTO-based TFTs, particularly V_TH_ and μ_FE_. To further clarify the origin of these electrical variations, the chemical bonding states of the ZTO, GZTO, and SZTO channel layers were analyzed by O 1s XPS, as discussed in the following section.

**Figure 5:**
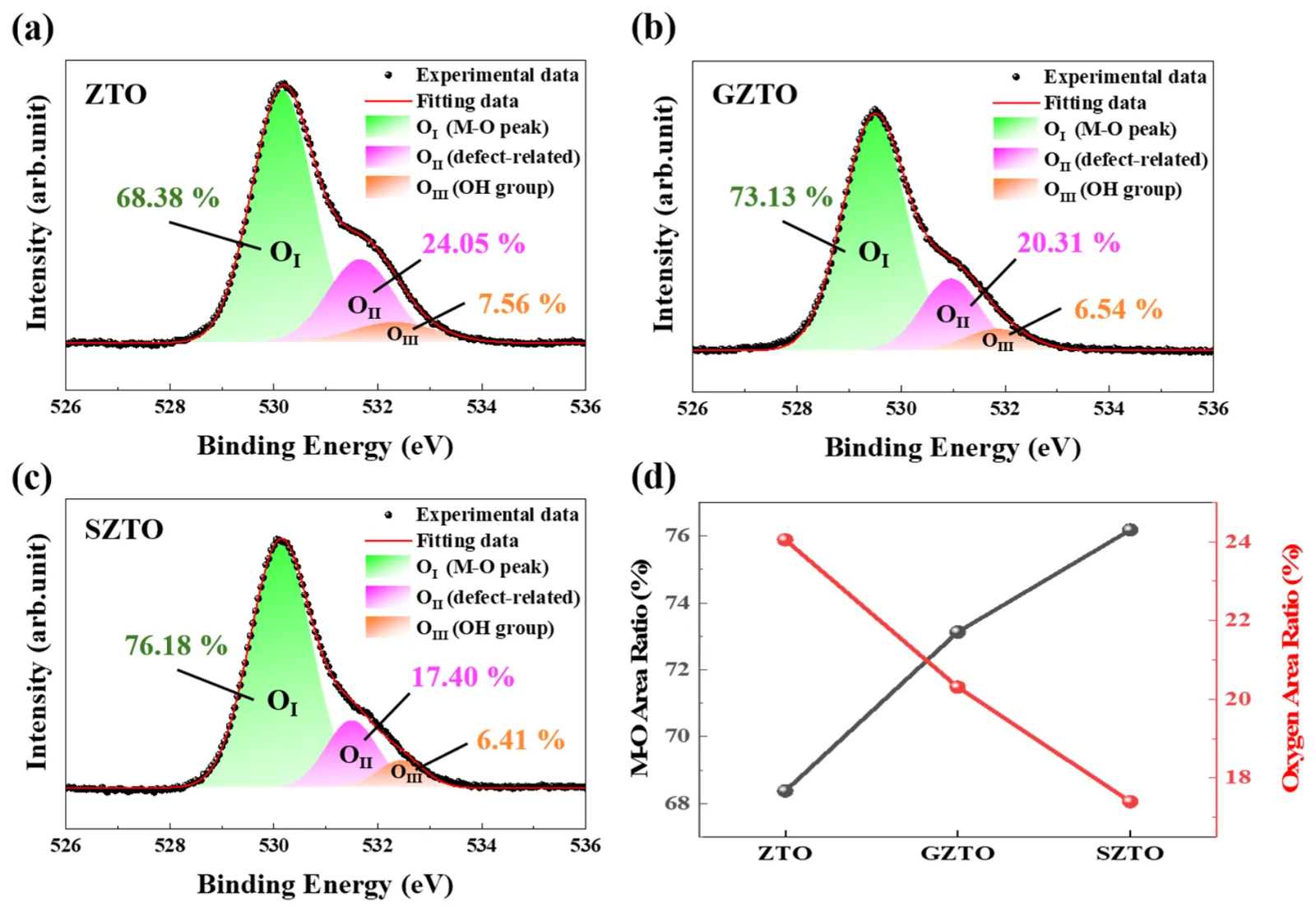
O 1s XPS spectra and deconvolution results of the (**a**) ZTO, (**b**) GZTO, and (**c**) SZTO thin films, and (**d**) extracted relative area ratios of the O_I_ and O_II_ components.

Figure 5 shows the O 1s XPS spectra and deconvolution results of the ZTO, GZTO, and SZTO thin films. The corresponding wide-scan XPS survey spectra are provided in Figure S1. Prior to peak deconvolution, all XPS spectra were calibrated using the carbon C 1s peak at 284.8 eV [24,25], and the high-resolution C 1s spectra used for the binding-energy correction are shown in Figure S2. The XPS-derived elemental compositions and O 1s peak-fitting parameters are summarized in Tables S1 and S2, respectively. The O 1s spectra were deconvoluted into three components: the O_I_ peak corresponding to metal–oxygen lattice bonds, the O_II_ peak associated with oxygen-deficient/surface-related bonding environments, and the O_III_ peak attributed to surface-adsorbed oxygen species, such as hydroxyl groups [26,27,28]. The O_II_ component may include contributions from different oxygen-deficient or surface-related chemical environments and therefore cannot be directly assigned to the concentration of oxygen vacancies.

Oxygen vacancies can exist in different charge states depending on their electron occupancy. Theoretical calculations for crystalline Zn_2_SnO_4_ have suggested that neutral oxygen vacancies can be energetically favorable under n-type conditions [29], whereas the local coordination disorder in amorphous oxide semiconductors can substantially modify the electronic structure of oxygen-deficiency-related defects [30]. Therefore, the dominant oxygen-vacancy charge state cannot be uniquely assigned in the present amorphous films, and conventional O 1s XPS cannot distinguish the individual populations of these charge states. Accordingly, the O_II_ variation is interpreted as a relative change in the overall oxygen-deficiency-related bonding environment rather than as selective suppression of a specific oxygen-vacancy charge state.

The relative area ratio of the O_II_ component was highest for the ZTO film, with a value of 24.05%, and decreased to 20.31% for GZTO and 17.40% for SZTO. Although the O_II_ component cannot be regarded as an absolute concentration of oxygen vacancies, its systematic reduction provides evidence for fewer oxygen-deficient bonding configurations in the ZTO-based films [31,32]. This result indicates that Ga and Si incorporation modified the oxygen-related bonding environment of the ZTO channel layers, with SZTO exhibiting the lowest O_II_ contribution.

The reduced O_II_ component is consistent with the positive shift in threshold voltage observed in Figure 3, where V_TH_ shifted from −2.48 V for ZTO to −1.32 V for GZTO and −0.61 V for SZTO. Since oxygen-deficiency-related defect states can contribute to donor-like states in oxide semiconductors, the decrease in the O_II_ component, together with the positive V_TH_ shift, is consistent with a reduced contribution of donor-like defect states [33,34]. Therefore, the XPS results support the conclusion that Ga and Si incorporation modifies the oxygen-related bonding environment in ZTO-based oxide semiconductors. Because conventional XPS predominantly probes the near-surface region, the O 1s results are used here as comparative chemical evidence rather than as a direct characterization of the buried gate-insulator/channel interface or the entire film bulk.

**Figure 6:**
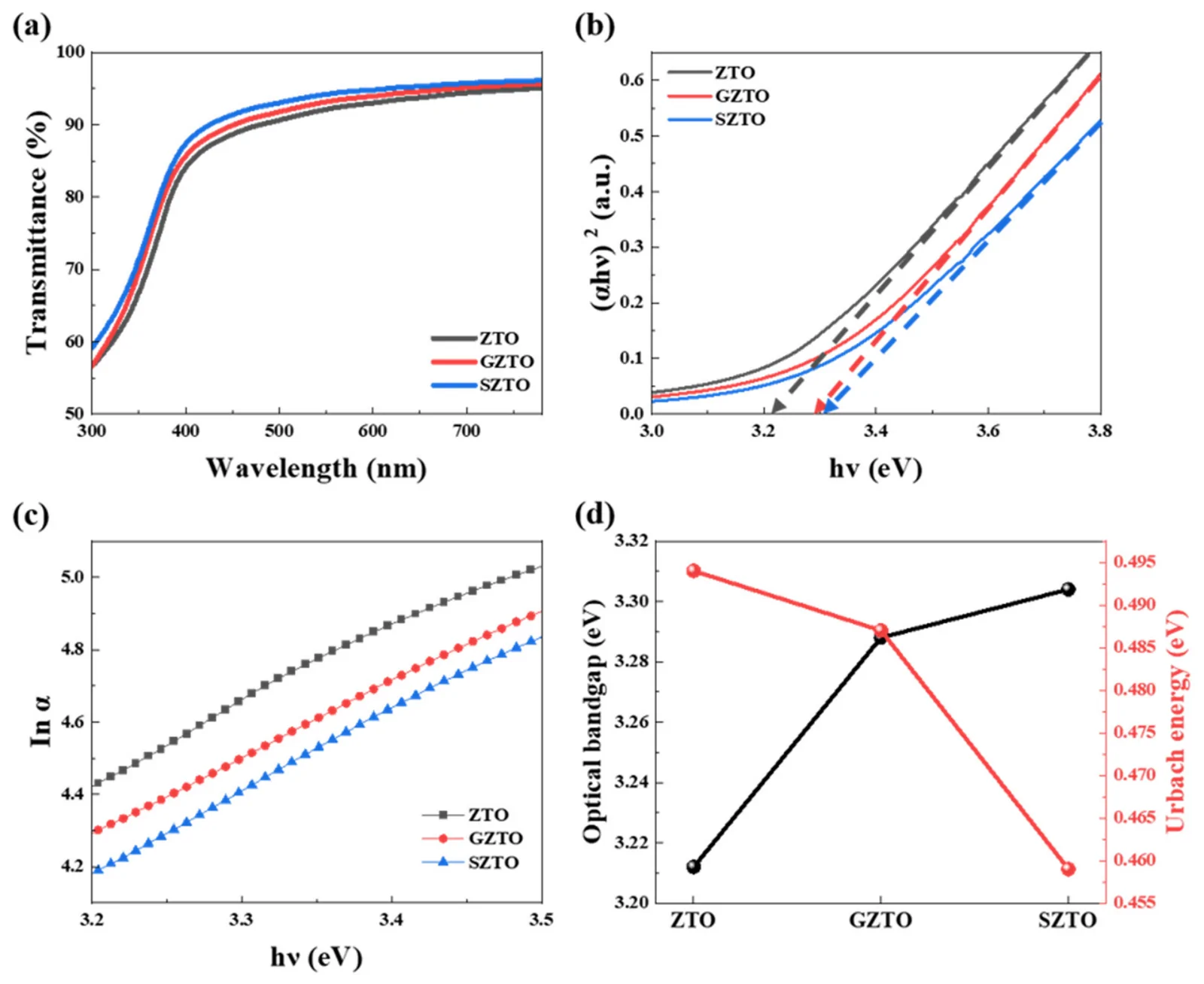
Optical properties of the ZTO, GZTO, and SZTO thin films: (**a**) optical transmittance spectra, (**b**) Tauc plots for optical bandgap extraction, (**c**) Urbach energy analysis, and (**d**) comparison of the optical bandgap and Urbach energy.

Figure 6 shows the optical properties of the ZTO, GZTO, and SZTO thin films. The optical transmittance spectra were measured using UV–visible spectroscopy, and the optical bandgap was extracted from the corresponding Tauc plots [35,36]. The absorption coefficient (α) was calculated from the measured optical transmittance using

$$\alpha =\frac{1}{d}ln(\frac{1}{T})$$

where T is the optical transmittance expressed as a fraction and d is the film thickness.

The optical gap was estimated using the Tauc relation for a direct allowed transition:

$${\left(\alpha hv\right)}^{2}=A(hv-E_{g})$$

where hv is the photon energy and A is a proportionality constant. The fitting region was selected from the continuous linear region in the steep absorption edge after departure from the lower-energy Urbach-tail regime [37]. As shown in Figure 6a, all the films showed high optical transmittance in the visible region. With the incorporation of Ga and Si dopants, the films exhibited a blue shift in their absorption edges, indicating an increase in the optical bandgap. This result is consistent with the Tauc plot analysis shown in Figure 6b, where the optical bandgap gradually increased from 3.212 eV for ZTO to 3.288 eV for GZTO and 3.304 eV for SZTO. This gradual increase suggests that Ga and Si incorporation affects the optical/electronic structure of the ZTO-based oxide films.

The widening of the optical bandgap may arise from both defect-related and chemical effects induced by Ga and Si incorporation. In amorphous oxide semiconductors, oxygen-deficiency-related states and structural disorder can generate sub-gap states and broaden the band-tail region [38]. Therefore, the decrease in the O_II_ contribution observed in the O 1s XPS analysis, together with the reduced Urbach energy, suggests a reduction in band-tail states that may contribute to the observed optical-gap widening. Because Ga_2_O_3_ and SiO_2_ possess wider bandgaps than ZTO, changes in the local bonding configuration associated with Ga–O and Si–O formation may also contribute to the observed absorption-edge shift. Although a Burstein–Moss contribution cannot be completely excluded, the positive V_TH_ shift and reduced I_ON_ do not support a substantial increase in free-electron concentration, suggesting that defect- and band-tail reduction is a major contributor to the observed optical-gap widening.

To further evaluate the degree of localized tail states, the Urbach energy was independently extracted from the linear region of ln(α) versus photon energy below the main absorption edge. The lower-energy region approaching the measurement limit and the higher-energy region associated with the onset of stronger absorption were excluded from the fitting. The ZTO film exhibited the highest Urbach energy of 0.494 eV, whereas the GZTO and SZTO films showed lower values of 0.487 and 0.459 eV, respectively. Since Urbach energy reflects the degree of structural disorder and localized tail states near the band edge, the reduced Eu values suggest that Ga and Si incorporation decreases defect-related localized states in the ZTO-based oxide films [39,40,41].

The SZTO film exhibited the lowest Urbach energy, consistent with the lowest O_II_ component observed in the XPS analysis. This agreement indicates that Si incorporation more effectively reduces oxygen-deficient bonding configurations and localized tail states, possibly owing to the stronger oxygen-binding character of Si. Therefore, the optical analysis supports the XPS results by showing that dopant incorporation reduces not only chemical defect-related components but also optically detectable band-tail disorder.

**Figure 7:**
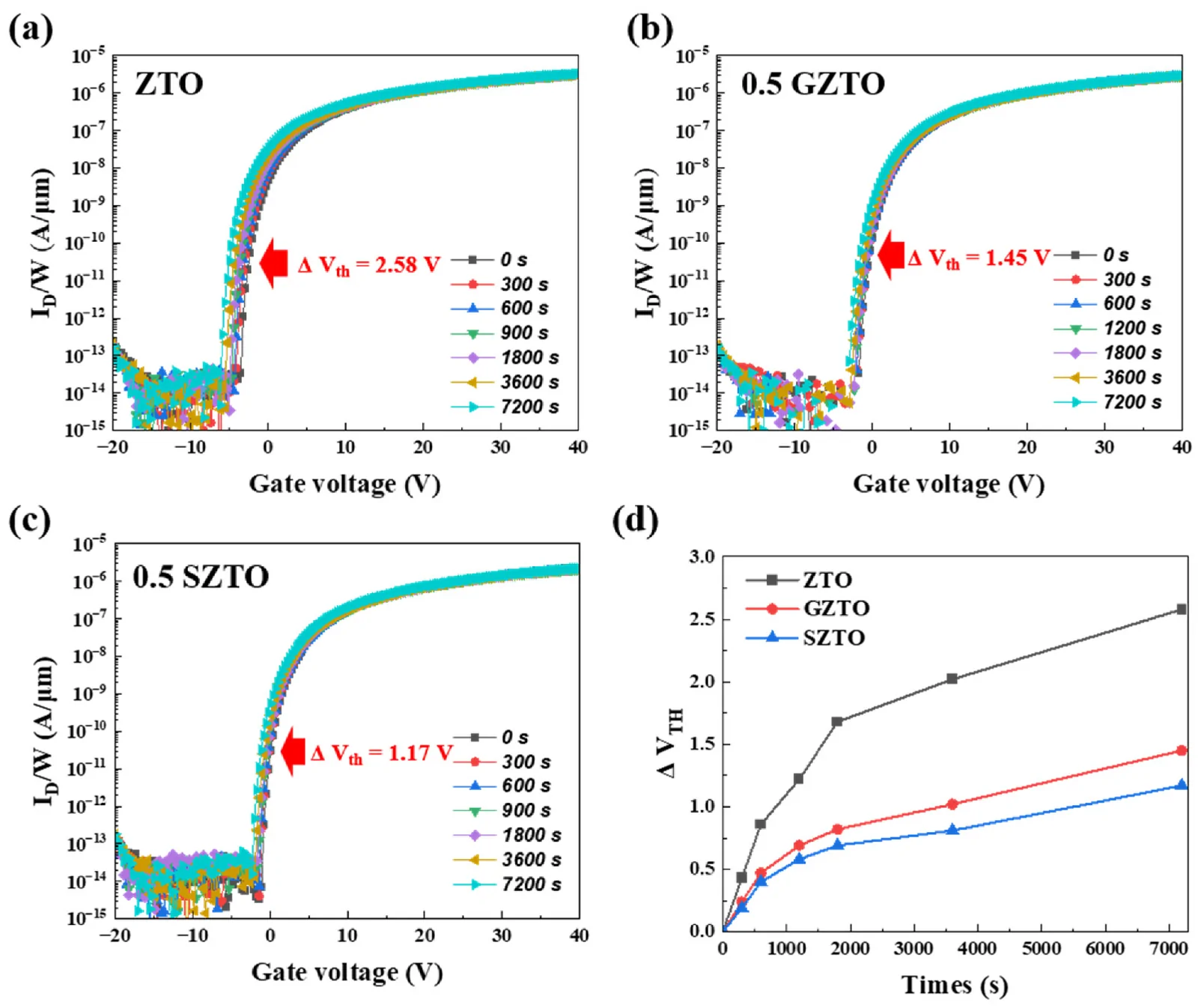
NBTS characteristics of ZTO, GZTO, and SZTO TFTs: (**a**–**c**) transfer characteristics under increasing stress time and (**d**) threshold-voltage shift as a function of stress time.

Figure 7 shows the NBTS stability characteristics of the ZTO, GZTO, and SZTO based TFTs. NBTS measurements were performed at a gate bias of V_G_ = −20 V and a temperature of 60 °C for up to 7200 s. After each stress duration, the transfer characteristics were measured to evaluate the threshold-voltage shift. During NBTS, all devices exhibited a negative shift in threshold voltage with increasing stress time [42,43]. However, the magnitude of ΔV_TH_ was clearly dependent on the channel composition. The ZTO TFT showed the largest ΔV_TH_ of 2.58 V after 7200 s, whereas the GZTO and SZTO TFTs showed reduced ΔV_TH_ values of 1.45 and 1.17 V, respectively.

Under negative gate bias and elevated temperature, the negative V_TH_ shift in amorphous oxide TFTs has been attributed to thermally activated charge trapping at defect states near the gate-insulator/channel interface, together with changes in the occupation of subgap states [44,45]. Elevated temperature can enhance these defect-mediated trapping processes, resulting in a progressively larger negative V_TH_ shift under stress. Although the present measurements do not independently distinguish the relative contributions of these mechanisms, the reduced O_II_ contribution and Urbach energy after Ga and Si incorporation are consistent with a lower overall contribution of oxygen-related and localized defect states that can participate in stress-induced trapping.

Accordingly, the smaller ΔV_TH_ observed in the GZTO and SZTO TFTs indicates improved resistance to bias-temperature-stress-induced instability. In particular, SZTO exhibited both the lowest ΔV_TH_ and the lowest OII contribution and Urbach energy, suggesting that Si incorporation more effectively suppresses defect-assisted charge trapping than Ga incorporation. A comparison of the electrical and defect-related parameters of the ZTO, GZTO, and SZTO TFTs is summarized in Table S3.

## 4. Conclusions

In this study, the effects of Ga and Si incorporation on the electrical characteristics, oxygen related defect states, optical properties, and NBTS stability of ZTO-based TFTs were systematically compared using an identical device platform. Both GZTO and SZTO TFTs exhibited positive threshold-voltage shifts compared with the ZTO TFT, consistent with a reduced contribution of donor-like defect states in the ZTO channel.

The O 1s XPS and optical analyses revealed that the defect-suppression effect was more pronounced in SZTO than in GZTO. The relative O_II_ contribution decreased from 24.05% for ZTO to 20.31% for GZTO and 17.40% for SZTO, while the Urbach energy also decreased from 0.494 to 0.487 and 0.459 eV, respectively. These results indicate that Si incorporation more effectively reduces the contribution of oxygen-deficient bonding environments and localized tail states in the ZTO-based oxide system.

The improved defect structure was accompanied by improved NBTS stability, where the threshold-voltage shift decreased from 2.58 V for ZTO to 1.45 V for GZTO and 1.17 V for SZTO. Overall, Ga incorporation preserved high field-effect mobility while moderately improving stability, whereas Si incorporation provided stronger defect suppression and superior NBTS stability with a moderate mobility trade-off. These findings demonstrate that dopant selection is a key factor in optimizing the balance between carrier transport and reliability in indium-free ZTO-based oxide TFTs.

## Figures and Tables

**Table 1:** Electrical characteristics of ZTO, GZTO, and SZTO TFTs. The values are presented as the mean ± standard deviation obtained from three devices for each composition, with one device measured from each of three independently fabricated batches.

Sample	V_TH_ (V)	I_ON_ (A)	I_OFF_ (A)	I_ON_/I_OFF_	μ_FE_ (cm^2^ V^−1^ s^−1^)	S.S.
ZTO	−2.48 ± 0.41	(6.99 ± 0.15) × 10^−4^	$(8.58\;\pm \;0.21)\times 10^{-12}$	$(8.14\;\pm \;0.27)\times 10^{7}$	27.1 ± 0.35	0.465 ± 0.012
0.5 GZTO	−1.32 ± 0.55	(6.77 ± 0.19) × 10^−4^	$(1.99\;\pm \;0.18)\times 10^{-12}$	$(3.41\;\pm \;0.32)\times 10^{8}$	27.6 ± 0.43	0.461 ± 0.018
0.5 SZTO	−0.61 ± 0.48	(5.11 ± 0.22) × 10^−4^	$(1.77\;\pm \;0.15)\times 10^{-12}$	$(2.89\;\pm \;0.26){\times 10}^{8}$	21.6 ± 0.61	0.437 ± 0.010

## Data Availability

The raw data supporting the conclusions of this article will be made available by the authors on request.

## Supplementary Materials

The following supporting information can be downloaded at https://www.mdpi.com/article/10.3390/mi17080985/s1, Figure S1: Wide-scan XPS survey spectra of ZTO, GZTO, and SZTO thin films; Figure S2: High-resolution C 1s XPS spectra of ZTO, GZTO, and SZTO thin films used for binding-energy correction; Table S1: XPS-derived elemental compositions of ZTO, GZTO, and SZTO thin films; Table S2: O1s XPS peak-fitting parameters of ZTO, GZTO, and SZTO thin films; Table S3: Comparison of electrical and defect-related parameters of ZTO, GZTO, and SZTO TFTs.
